# Control of human Q fever by vaccination: the journey to Q-VAX and beyond

**DOI:** 10.1128/iai.00280-25

**Published:** 2025-11-06

**Authors:** Robert A. Heinzen, Kathleen N. Pierce, Daniel E. Voth, John Stenos, Stephen Graves, Carrie Mae Long

**Affiliations:** 1 Laboratory of Bacteriology, Division of Intramural Research, National Institute of Allergy and Infectious Disease, National Institutes of Health 2511, Hamilton, Montana, USA; 2 Department of Microbiology and Immunology, University of Arkansas for Medical Sciences 12215, Little Rock, Arkansas, USA; 3 Australian Rickettsial Reference Laboratory, Barwon Health 12215, Geelong, Victoria, Australia; University of California Merced, Merced, California, USA

**Keywords:** *Coxiella burnetii*, Q fever, Q-VAX, vaccine, bacterial vaccine, history, human vaccination

## Abstract

Current vaccine development efforts encompass diverse and innovative approaches; however, studies with basic vaccines (e.g., whole cell inactivated) continue to inform these efforts. Perhaps the world’s most effective and durable human vaccine, the query (Q) fever vaccine known as Q-VAX, can trigger a severe post-vaccination hypersensitivity reaction, precluding its widespread deployment. The history of Q fever vaccine development serves as a rich example of the power of scientific collaboration, elegant experimentation, and the study of adverse post-vaccination events. Here, we review the history of Q fever vaccine development, profiling seminal studies while also relating this information to modern vaccine development efforts.

## *COXIELLA BURNETII* AND QUERY FEVER

*Coxiella burnetii*, the bacterial causative agent of human query (Q) fever, is a highly infectious, zoonotic pathogen with livestock serving as the primary reservoir (1). Due to pronounced extracellular stability, the debilitating nature of acute Q fever, and an aerosol infectious dose approaching one organism, *C. burnetii* has been tested as a biological weapon (2, 3) and is considered a bioterrorism threat (4). The pathogen has a notorious history of causing laboratory-acquired infections (LAIs) (5) and is an occupational hazard for individuals in contact (direct or indirect) with ruminant hosts and their by-products, including personnel in animal husbandry, meat processing, dairy, and veterinary professions (6–10). Furthermore, military personnel deployed in endemic regions are at increased risk of infection (11).

Q fever is a complex illness characterized by heterogeneous clinical presentations and outcomes. Acute Q fever ican be a debilitating influenza-type illness that generally resolves without complications after several weeks (1). Of elevated concern are the roughly 15%–20% of infected people that experience serious long-term sequelae following symptomatic or asymptomatic infection, including Q fever fatigue syndrome (15%), chronic endocarditis, and other vascular disease (5%) (12–15). The disease is nearly 100% preventable using a vaccine composed of formalin-inactivated, whole cell bacteria (16).

In 1935, a field study of Rocky Mountain spotted fever (RMSF) was being conducted at Rocky Mountain Laboratories (RML), National Institutes of Health (NIH) in Hamilton, MT, USA. *Dermacentor andersoni* ticks collected in Nine Mile Valley, MT, were fed on guinea pigs to isolate new strains of *Rickettsia rickettsii*, the cause of RMSF. Interestingly, one animal developed a febrile illness that did not mimic RMSF clinical presentation (17). Termed the “Nine Mile agent,” investigators Gordon Davis and Herald Cox demonstrated that the pathogen could be serially passaged in guinea pigs but did not grow in axenic culture (17).

Coincidentally, Edward Derrick described Q fever as a clinical entity in 1937 in association with an outbreak of febrile illness in nine abattoir workers in Brisbane, Australia (18). Patients suffered from fever lasting 7–24 days with additional symptoms including headache, joint pain, and anorexia. Derrick transferred the disease to guinea pigs by injection with the patient’s blood, but attempts to characterize the infectious agent were unsuccessful (18). For assistance, Derrick forwarded infected guinea pig tissue to Frank (Macfarlane) Burnet at the Walter and Eliza Hall Institute in Melbourne, who, along with Mavis Freeman, reproduced the characteristic febrile reaction in guinea pigs (19). Serum of convalescent Q fever patients agglutinated suspensions of clarified tissue from these animals. Furthermore, stained smears of infected spleen tissue contained small microorganisms that appeared to be of a “rickettsial nature.” As experienced by Derrick, attempts by Burnet to cultivate the agent on artificial media also failed.

In 1938, at RML, a chance infection of a visiting laboratory administrator (Rolla Dyer, NIH Director) by the Nine Mile agent revealed a connection with Q fever (20). Dyer showed clinical symptoms remarkably akin to the newly described Q fever disease in Australia (21). Subsequently, cross-protection studies in guinea pigs established that the Q fever and Nine Mile agents were closely related, if not the same pathogen (22). Eventually, this novel intracellular bacterium was placed in a new genus, *Coxiella*, and species, *burnetii*, in honor of Cox and Burnet, respectively (23).

## EARLY VACCINE HISTORY

Soon after the discovery of *Coxiella burnetii* as the causative agent of Q fever, researchers demonstrated proof of principle that chemically fixed, whole cell *C. burnetii* was immunogenic. Crude vaccines prepared from fixed, infected spleen homogenates (guinea pig or mouse), chick embryo tissue, or ticks were highly protective in guinea pig challenge models (24, 25). Initial human Q fever vaccines were composed of semi-purified *C. burnetii* from yolk sac membranes of infected embryonated hens’ eggs, a tissue that robustly supported *C. burnetii* growth (26). Vaccines from egg-adapted strains were developed to protect laboratory workers and associated staff, field units, and frequent laboratory visitors (5, 27–29). Indeed, laboratory-acquired Q fever infections occurred coincident with the original isolation of *C. burnetii* (21, 30) and continued to plague research facilities thereafter, including the NIH (27, 29, 31), a US Army Research Laboratory (5), Bristol University (32), and the University of Cambridge (33).

**Fig 1:**
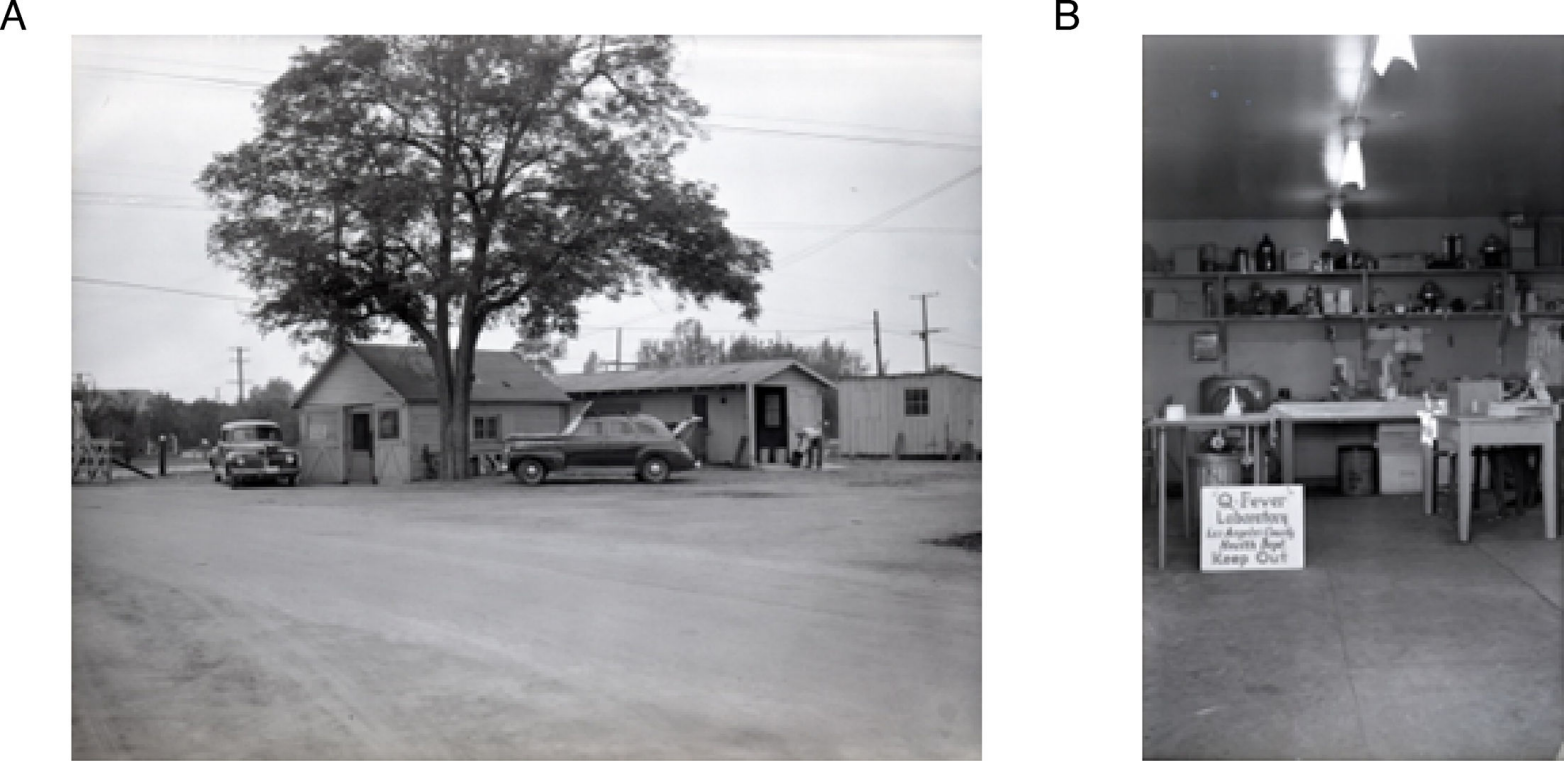
Historical Q fever investigations by the Viral and Rickettsial Disease Laboratory of the California State Department of Public Health. The Los Angeles County Health Department Q fever field laboratory in Hondo, California, 1948 (**A and B**). Photos courtesy of National Institute of Allergy and Infectious Diseases.

Early Q fever whole cell vaccines (WCVs) were primarily prepared by the method of Smadel and were referred to as “Smadel-type” vaccines (28, 29, 33, 34). Smadel-type vaccines were used extensively in the 1960s to protect at-risk laboratory workers (28, 29, 33, 35, 36). The Smadel preparation began with a 20% suspension of infected yolk sac in physiologic saline (0.15 M NaCl) and was first adjusted to 0.5% formaldehyde. The suspension was then stored at 4° for 3–5 days to inactivate *C. burnetii*, diluted with saline to a 10% solution, then extracted with ether. Ether extraction was critical to generate a high-potency vaccine with reduced egg allergens, a procedure used to purify the epidemic typhus bacterium, *Rickettsia prowazekii*, for vaccine use (37). Little ether-soluble antigenic material was released by *C. burnetii* (38). After low-speed centrifugation of the mixture, the aqueous phase containing *C. burnetii* was collected and used directly as a vaccine. A slight variation of this method was later used by Smadel et al. (28, 34) and Berman et al. (39). In 1947, Smadel conducted the first human Q fever vaccination trial with 39 volunteers (34). The Smadel-type vaccine was considered efficacious due to positive human serological responses and protection against disease in a parallel guinea pig study. Another early study involved employees of the Viral and Rickettsial Disease Laboratory of the California State Department of Public Health, who were charged with investigating large dairy farm-associated outbreaks of Q fever in Los Angeles County (Fig. 1A and B) (28). Fifty at-risk workers were vaccinated in 1948–1949 with a Smadel-type vaccine. Again, vaccine efficacy was implied based on positive serological responses and no cases of clinical Q fever among vaccinees (Fig. 1C).

## HUMAN CHALLENGE

Following early Smadel-type WCV administration, a seminal event in Q fever vaccine development occurred in 1955, when a direct demonstration showed that a Smadel-type WCV protected humans from aerosol *C. burnetii* challenge (40–43). These trials were conducted with volunteers participating in Project Operation Whitecoat (POW), a component of the US Army’s biological warfare defense program that ran from 1954 to 1973 (44). A WCV was made with the Henzerling strain (after 22 egg passages) that was originally isolated from the blood of a US soldier in Italy during World War II (45, 46), and the challenge was conducted with the California AD strain (after 8 egg passages) originally isolated from cow’s milk in California (46, 47). POW volunteers were immunized with three 1 mL doses of 10 complement fixation units (CFU)/mL and were protected from challenge with as high as 150,000 50% guinea pig infectious doses (ID_50_). Seminal findings of the project were (i) the human aerosol infectious dose of *C. burnetii* approached one organism; (ii) the human aerosol infectious dose directly correlated with the incubation period; (iii) human vaccination protected against high-dose aerosol challenge; (iv) human vaccination was protective if administered 24 h after low-dose challenge; (v) the disease in guinea pigs, infected via the intraperitoneal or aerosol route, was comparable to that of humans; and (vi) the infectious dose/fever response of guinea pigs was similar to humans, validating the guinea pig as a physiologically relevant rodent model of Q fever. Despite the valuable data produced by POW, the ethical implications of this project have been questioned, ranging from its perception as a “model of ethical human experimentation” (48) to an ethically dubious pursuit that would not pass modern ethical standards (49).

## IMPROVED *C. BURNETII* PURIFICATION PROTOCOLS

Characterization of bacterial stocks and the potency of early vaccines were measured by serological detection of *C. burnetii* using a complement fixation (CF) assay that quantified CFU (36). The term “phase variation” was adopted to describe the peculiar serological behavior of *C. burnetii* and will be discussed later in this review. In short, phase I refers to virulent *C. burnetii* strains, and phase II refers to avirulent *C. burnetii* strains (50, 51). Ormsbee and colleagues carefully established relationships between CFU and purity, infectivity, and vaccine potency of *C. burnetii* suspensions derived from infected yolk sacs (52–54). CFU per milligram total nitrogen (N) was considered to be a relative measure of *C. burnetii* purity, and stocks with values as high as 6,000 CFU/mg N were achievable with Smadel-type preparations (52). Nevertheless, a general concern with vaccines derived from organisms propagated in eggs was that contaminating egg protein might induce anaphylaxis in allergic individuals (36, 54, 55). To address this concern, Ormsbee developed a protocol that dramatically lowered egg protein contamination by incorporating a continuous-flow centrifugation step to sediment *C. burnetii* organisms from soluble yolk sac material that were then ether-extracted (54). This procedure produced a vaccine containing dramatically less yolk sac protein (albumin) than a Smadel-type vaccine with serologically undetectable egg component contamination (36, 54). Lackman and co-workers (36) then established the recommended minimum requirements for both preparation and standardization of an Ormsbee-like Q fever vaccine based on sufficient purity and potency. Vaccine requirements included a concentration of 10 CFU/mL and a purity of at least 1,000 CFU/mg N. Suitable preparations were not allowed to contain over 40 CFU/mL of serologically detectable egg albumin; thus, a vaccine dose of 10 CFU/mL in physiologic saline was recommended. The high purity of bacteria obtained using the Ormsbee method eventually allowed the use of *C. burnetii* dry weight in vaccine dosing, with 30 µg established as an effective human dose (54, 56, 57). Centrifugation of mechanically disrupted and partially clarified infected yolk sac homogenates to equilibrium in gradients of sucrose or Renografin (diatrizoate meglumine and diatrizoate sodium) also purified *C. burnetii* to near homogeneity (58–61). Dry weight could be established by simply weighing purified and dried bacteria or by converting *C. burnetii* particle counts to dry weight (62).

## DEVELOPMENT OF REACTOGENICITY AND HYPERSENSITIVITY TESTING

Early vaccination protocols during preliminary testing were infamous for causing a severe local reaction at the vaccination site. Reactions could range from a small, indurated mass to a large, discharging sterile abscess that required surgery for resolution (28, 29, 34, 36, 40, 63–65). For example, of 98 individuals vaccinated at RML in 1958, 21 showed evidence of abscess formation after a single dose of vaccine (29, 63). It became clear that serious reactions were more common among individuals who received multiple vaccinations (28, 40, 63, 64). Reactions after a single vaccination were indicative of pre-existing immunity resulting from either symptomatic infection or asymptomatic exposure (36). Owing to the inactivated nature of the vaccine, most experts believed that repeated vaccination was required to maintain immunity (28, 34, 63), and the single-dose efficacy of this vaccine would not be ascertained until later.

Reactogenicity of Q fever vaccines prompted development of protocols to screen for previous exposure, a finding that would preclude vaccination. Several serological techniques were developed with markedly different sensitivities (66). A landmark study by Lackman et al. established a hypersensitivity skin test to supplement serology in identifying pre-exposed individuals (64). A pilot study using a Smadel-type vaccine was conducted on 55 employees at RML, in which a 0.1 mL dose containing 0.01 CFU of *C. burnetii* derived as a 1:100 dilution of WCV was injected intradermally. A positive reaction was defined as a region of well-defined erythema of 3 mm or more in diameter that was generally most pronounced at 1 week post-inoculation. This study revealed a correlation between positive serology (by CF assay) and skin test reactivity. The study also introduced rabbits and guinea pigs as animal models for hypersensitivity testing. A large field study was then conducted around Boise, ID, USA, a geographic region with high numbers of *C. burnetii*-infected cattle, to compare testing strategies. Of 306 people tested, 104 (34%) showed evidence of past infection by CF testing. Of 93 skin test-positive individuals, only 62 (67%) were also positive by CF assay. It was noted that the lack of concordance was likely due to the transient nature of specific antibodies (65). Moreover, several individuals positive by CF assay were negative by skin test, and this group was dominated by individuals with a low CF titer of 1:8. Ultimately, skin testing was determined to be preferable to serology in determining past *C. burnetii* infection*,* but confidence was gained when using both tests prior to vaccination. Later studies by Lackman and colleagues (67) dropped transient erythema in favor of induration as an indicator of past exposure.

## EARLY EFFICACY TESTING OF WCV FRACTIONS

The availability of a hypersensitivity skin test allowed researchers at RML to investigate whether fractions could be derived from *C. burnetii* that conferred protection without reactogenicity. Anacker et al. (68) compared trichloroacetic acid (TCA) extract from the Ohio 314 phase I strain to whole cells using guinea pigs and sensitized rabbits as challenge and skin test models, respectively. On a weight basis, TCA extract, which retains phase I reactivity, displayed approximately 10% and 1% of the potency demonstrated by whole cells as vaccine and skin test antigens, respectively. Quantitative differences in protection and reactivity suggested that the two were associated with distinct immunological pathways. A subsequent study showed that phenol extraction of residual protein from TCA extract completely abolished skin reactivity. The authors concluded that phenol treatment removes a carrier protein from a polysaccharide cell wall component, rendering the polysaccharide moiety haptenic. Furthermore, the authors proposed that polysaccharide was an essential component of the phase I antigen (69).

**Fig 2:**
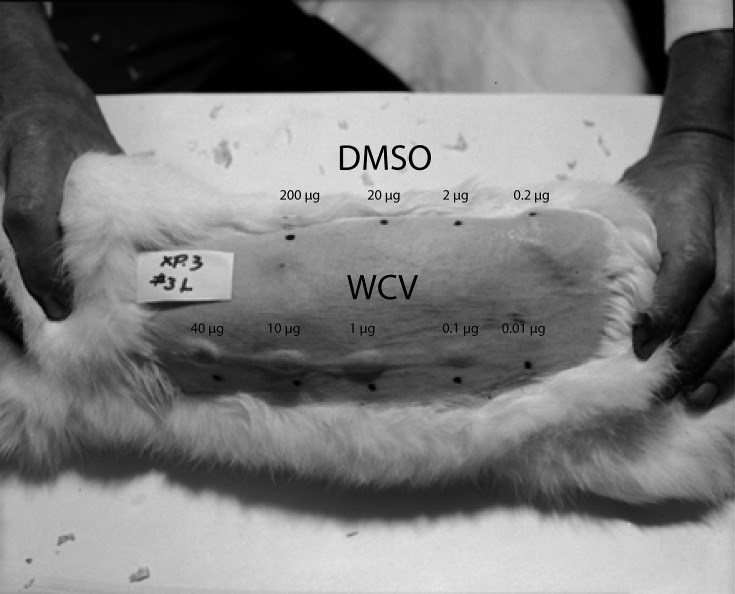
Rabbit hypersensitivity model for testing antigenic WCV fractions. Ohio 314 WCV and DMSO extracts were injected intradermally into a rabbit sensitized with Ohio 314. (Left to right) The upper row of ink spots denotes DMSO extract in doses of 200.0, 20.0, 2.0, and 0.2 µg, while the lower row denotes WCV in doses of 40.0, 10.0, 1.0, 0.1, and 0.01 µg. A small hypersensitivity lesion (edema and induration) is seen only at the 200 µg DMSO extract injection site, while lesions extend down to the 0.1 µg WCV injection site. Photo courtesy of the National Institute of Allergy and Infectious Diseases.

Meanwhile, Ormsbee and colleagues (70) tested dimethyl sulfoxide (DMSO) extracts of phase II Nine Mile and phase I Ohio 314 *C*. *burnetii* strains in guinea pig and rabbit models. When guinea pigs were challenged with the California 16 phase I strain, 40 µg of DMSO extract of Ohio 314 conferred protection equal to 4 µg of whole cell bacteria. DMSO extracts of phase II Nine Mile were considerably less effective, with extracts being at least 58 times less potent. Correspondingly, a WCV composed of Nine Mile phase II was at least 100× less potent than a WCV made from Ohio 314. In sensitized rabbits, the least amount of DMSO extract of Ohio 314 required for a hypersensitivity skin reaction was 200 µg, while significant hypersensitivity lesions were observed with 0.1–1.0 µg of WCV (Fig. 2).

The collective work of Anacker et al. (68) and Ormsbee et al. (70) regarding *C. burnetii* fractionation strongly indicated that lipopolysaccharide (LPS) comprised a critical protective antigen of phase I *C. burnetii* and was also responsible for phase I serological reactivity. Indeed, Abinanti and Marmion (71) had previously suggested that antibody to the phase I antigen was associated with immunologic protection. It was conversely surmised that deleterious skin reactions were associated with a separate proteinaceous antigen (72). Several studies suggested that the reactogenic component was proteinaceous, while an important protective component was LPS *O*-antigen polysaccharide (73). For example, the California AD phase II strain had similar skin testing potency as the Ohio 314 phase I strain (36). Comparable reactogenicity by phase II and phase I WCVs has also been demonstrated by contemporary studies (74).

Concurrent with Ormsbee et al.’s work (70), Brezina and Urvologyi (75) of the Slovak Academy of Sciences also demonstrated that phase I, but not phase II, antigen could be removed from *C. burnetii* by TCA extraction. Phase I reactivity of both whole cell organisms and TCA extracts was destroyed if either was treated with periodate, again pointing to lipopolysaccharide as the reactive phase I component. After demonstrating protection and low reactogenicity in animals, the TCA extract was tested as a vaccine on 46 human Q fever laboratory workers with potential exposure to *C. burnetii*, who were also serologically and skin test negative. No infections occurred in individuals vaccinated once or twice, while five infections occurred among the unvaccinated control group. Serious local reactions were not observed, and the vaccine was noted as “promising” (76). The TCA extract or “chemovaccine” was then examined in a large immunization study in the former Czechoslovakia in 1977, 1978, and 1980 on persons artificially exposed to *C. burnetii* (77). A vaccine dose consisted of TCA-soluble material from 1 mg of dried *C. burnetii*. Forty percent of vaccinees suffered allergic skin reactions, although progression to a sterile abscess was not observed. While protection was not evaluated, the lack of infections among the study cohort suggested efficacy (29). In summary, early extract or “split” *C. burnetii* vaccines conferred moderate protection with reduced reactogenicity relative to WCVs in pre-exposed individuals. These auspicious results led to more extensive investigations of the delipidated residue remaining after chloroform:methanol treatment of *C. burnetii* as an extract vaccine.

## EARLY OBSERVATIONS OF *C. BURNETII* PHASE VARIATION SHAPED VACCINE DEVELOPMENT

Early after the discovery of *C. burnetii*, it became evident that CF titers could differ dramatically based on the strain used as test antigen (78). Paradoxically, this occurred despite strains showing complete reciprocal cross-immunity in animal challenge studies (78). Stoker (50, 51) recognized that serological reactivity was not associated with strain identity *per se*, but rather with strain egg adaptation that he speculated might result in a change in antigenic structure. Fiset and Stoker (79, 80) observed that both early (<20 days) and late (>20 days) sera from infected guinea pigs reacted with bacteria in phase II, while only late sera reacted with bacteria in phase I. Serological reactivity was associated with culture history; i.e., primary isolates always displayed phase I reactivity that then converted to phase II reactivity after approximately 10 egg passages. Fiset (79) suggested that, although low-passage *C. burnetii* produced both phase I and phase II antigens, phase II antigens were “masked” by phase I antigens, thereby inhibiting antibody interactions *in vitro*. He further speculated that phase I and phase II antigens were “poor” and “good” antigens, respectively. The poor phase I antigen was proposed to be a polysaccharide, a hypothesis buttressed by Fiset’s preliminary work showing that potassium periodate treatment of phase I *C. burnetii* unmasked the component responsible for phase II reactivity (81). He further posited that, due to their natural occurrence, phase I organisms were more virulent than phase II bacteria and that vaccines made with phase I organisms would be more efficacious than the currently used vaccines made with egg-adapted phase II strains. As further discussed in this review, Fiset had uncanny foresight in speculating that phase variation was a critical parameter in the preparing diagnostic antigens and optimizing WCV vaccine potency. Indeed, Chen et al. have further demonstrated that phase variation plays an important role in the immune response to WCVs and specifically identified that cell-mediated immunity is dependent on the LPS phase of the WCV (82).

## *C. BURNETII* PHASE VARIATION AND VACCINE EFFICACY

Vaccine subfraction studies correlated phase I reactivity with elevated protection and provided an initial demonstration that a WCV was optimally potent if derived from phase I bacteria (68, 70, 75, 76). Subsequently, Ormsbee and co-workers (56) thoroughly examined the influence of LPS phase on vaccine potency using isogenic phase I and phase II variants of the Nine Mile, Henzerling, and Ohio isolates as vaccine strains. On average, phase I WCVs were 154-fold more potent than phase II WCVs (46, 56). Importantly, they also demonstrated that vaccine potency was influenced by the phase of the challenge strain; i.e., no difference in potency was observed if phase I or phase II vaccinated animals were challenged with phase II bacteria (56). This result was attributable to a severe attenuation in virulence associated with the phase II antigenic shift, as shown by direct comparisons of isogenic phase I and phase II variants of the Grita (M44) (83), Nine Mile (57, 62, 81), and Henzerling (84) strains in guinea pig challenge models. Interestingly, two experimental vaccines based on live phase II organisms were tested in humans. A small trial with live Nine Mile phase II (after 88 egg passages) and human volunteers showed protection against low, but not high, dose aerosol challenge (57). The M44 phase II variant of the Grita strain (after 44 egg passages) was used in Russian field trials (83, 85); however, human challenge data were lacking. The proven efficacy of killed vaccines of phase I organisms, combined with the possibility of reversion to virulent phase I organisms (85), eliminated viable phase II vaccines from serious consideration. Indeed, subsequent studies of M44 demonstrated long-term survival in guinea pigs, leading to vaccine-related hepatitis, splenitis, and myocarditis (86, 87).

As noted by Fiset (57), early Q fever vaccines were typically made from egg-adapted strains of *C. burnetii* that had likely converted, at least in part, to phase II bacteria (34, 40, 72, 88). Of note, the original POW human volunteer studies used a WCV made from the Henzerling strain after 22 egg passages (40, 41). Using a new equilibrium density gradient sedimentation method that separated phase I and phase II cells, RML researchers determined that the Henzerling seed material used in vaccine production was predominantly in phase II, and the California AD strain used for challenge was a mixture of phase I and phase II cells (88, 89). Collectively, *C. burnetii* phase variation findings caused a major reassessment of Q fever vaccine design with the phase II Henzerling vaccine already in a large field study (35). Ormsbee et al. (46) converted the Henzerling phase II seed stock to phase I by guinea pig passage, and a new seed stock was prepared by the Division of Biologics Standards, NIH, that was used in the preparation of a Henzerling phase I vaccine lot (88). Between 1966 and 1968, a single 30 µg subcutaneous dose, based on Ormsbee et al. (56), was administered to 64 human volunteers, of whom 13 were challenged 10 months later via the aerosol route with 3,000 guinea pig ID_50_ of a phase I California AD strain (57). Following the challenge, no vaccinees developed signs of illness. Contributing to the efficacy of earlier phase II WCVs were the multiple-dose vaccination regimes with individual primary and booster doses predicted to contain approximately 100 µg of *C. burnetii* (46). This large amount of antigen, while conferring protection, also likely primed individuals for aggravated delayed-type hypersensitivity reactions at the vaccination site, as phase II bacteria retain antigens that elicit a skin hypersensitivity response (36).

It is now clear that LPS *O-*antigen is the primary seroreactive and structural antigen that distinguishes phase I from phase II bacteria (77, 90, 91). Cumulatively, biochemical analysis (90, 92–94), virulence testing (62, 73, 95), vaccine evaluation (56, 74, 96), and genomic sequencing (94, 97, 98) of the isogenic Nine Mile phase I and Nine Mile phase II strains implicate LPS as the sole factor responsible for the disparate virulence and vaccine potency of the two strains. Surface-exposed *O-*antigen chains become increasingly truncated during *in vitro* passage, culminating in an LPS consisting of lipid A and inner core sugars (93, 94, 99). Genetic lesions responsible for this smooth-to-rough LPS transition can range from non-reversible, large deletions (>25 kb) to revertible point mutations in genes responsible for *O*-antigen and/or outer core biosynthesis (94, 100). Consequently, high-passage phase II variants that lack *O*-antigen altogether are highly attenuated in a guinea pig infection model (62, 73, 74, 95). Phase I *C. burnetii* virulence is related to the ability of full-length LPS to sterically inhibit (i) antibody binding to surface proteins (phase II antigens) (101); (ii) complement deposition (102); and (iii) interaction of pathogen surface ligands, such as lipoproteins, with phagocyte toll-like receptors (103). Lacking this virulence determinant, phase II bacteria were predicted to be unable to survive passage through an immunocompetent animal. Consequently, *C. burnetii* stocks can be “cleansed” of phase II bacteria by animal passage as was done with the Henzerling vaccine strain (46). Furthermore, a modern *in vitro* “cleansing” method was introduced by Long et al. involving passage of *C. burnetii* on confluent Vero cells (104). Of note, a novel mechanism of phase II to intermediate LPS elongation was identified in 2024 involving a 3 bp, slipped strand mutation in the gene *cbu0533*, resulting in enhanced virulence of the NMII strain (105). Ultimately, the precise contribution of LPS *O*-antigen to vaccine efficacy is unknown. Abinanti and Marmion (71) demonstrated a correlation between the ability of immune serum to passively protect mice and the amount of antibody against phase I antigen present in serum. The protective mechanism of anti-*O*-antigen antibody is unknown, but it does not appear to require complement or Fc receptor functions (106).

## DEVELOPMENT OF THE Q-58A VACCINE AT RML

**Fig 3:**
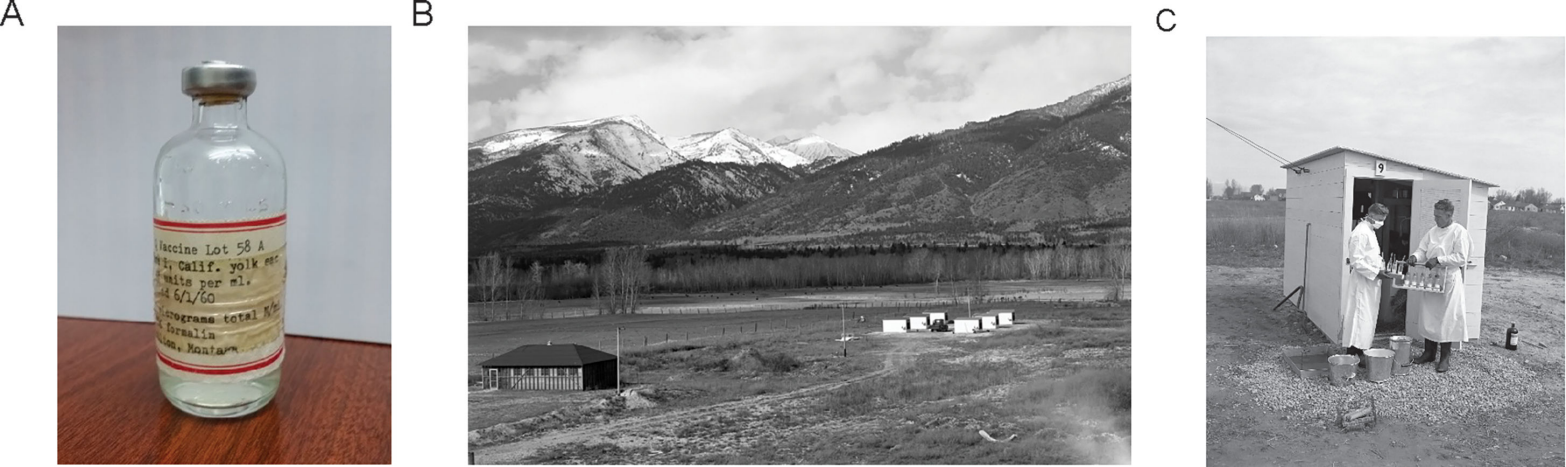
Q fever vaccine development and dairy cow testing for *C. burnetii* at RML. (**A**) Vial of Q-58A developed and tested at RML. (**B and C**) Individual barns used for *C. burnetii* detection in dairy cows. Photos circa 1948 by Kramis (courtesy of the National Institute of Allergy and Infectious Diseases).

Criteria established by Lackman and co-workers were used to produce the Q fever vaccine Q-58A (Fig. 3A) (36, 54). From 1960 to 1983, a clinical trial of this Ormsbee-type vaccine was conducted at RML (29). Q58-A was given the investigational new drug number Biological-Based-Investigational New Drug-26 (BB-IND-26) by the Division of Biologics Standards, NIH (now the Center for Biologics Evaluation and Research, US Food and Drug Administration). This WCV was manufactured in 1960 and stored as an aqueous solution containing 22 µg/mL with a single dose of 1 mL. Amazingly, when stored at room temperature, the vaccine did not lose potency when tested in a standard guinea pig protection test in 1970, 1980, and 1984 (29). Further demonstrating the stability of Q fever WCV, autoclaving for 15 min did not affect vaccine potency (70). Over 20 years, 398 RML employees were skin tested using California AD phase I strains. The skin test consisted of a 100-fold dilution of the vaccine dose delivered intradermally and screened for edema and induration after 1 week (107). Skin-reactive individuals with pre-existing immunity were more likely to have been in close contact with relevant animal reservoirs and/or were employees of Q fever research laboratories. Indeed, over a 20-year period of routine serological screening and vaccination of employees at RML, 79 (20%) out of 398 were skin test-positive. This high exposure rate was due to both employment where LAIs were common and/or the rural character of the area that provided ample opportunity for contact with zoonotic reservoirs (29). Accordingly, LAI associated with infected livestock on the RML campus (Fig. 3B and C) and at the University of Bristol (108), among others, has been documented. Around the same time as the development of the BB-IND-26 vaccine, the National Drug Company developed a similar vaccine based on the Henzerling strain that was used in the US Army’s special immunization program (Fig. 3D) (109).

## THE RML: Q-VAX CONNECTION

**Fig 4:**
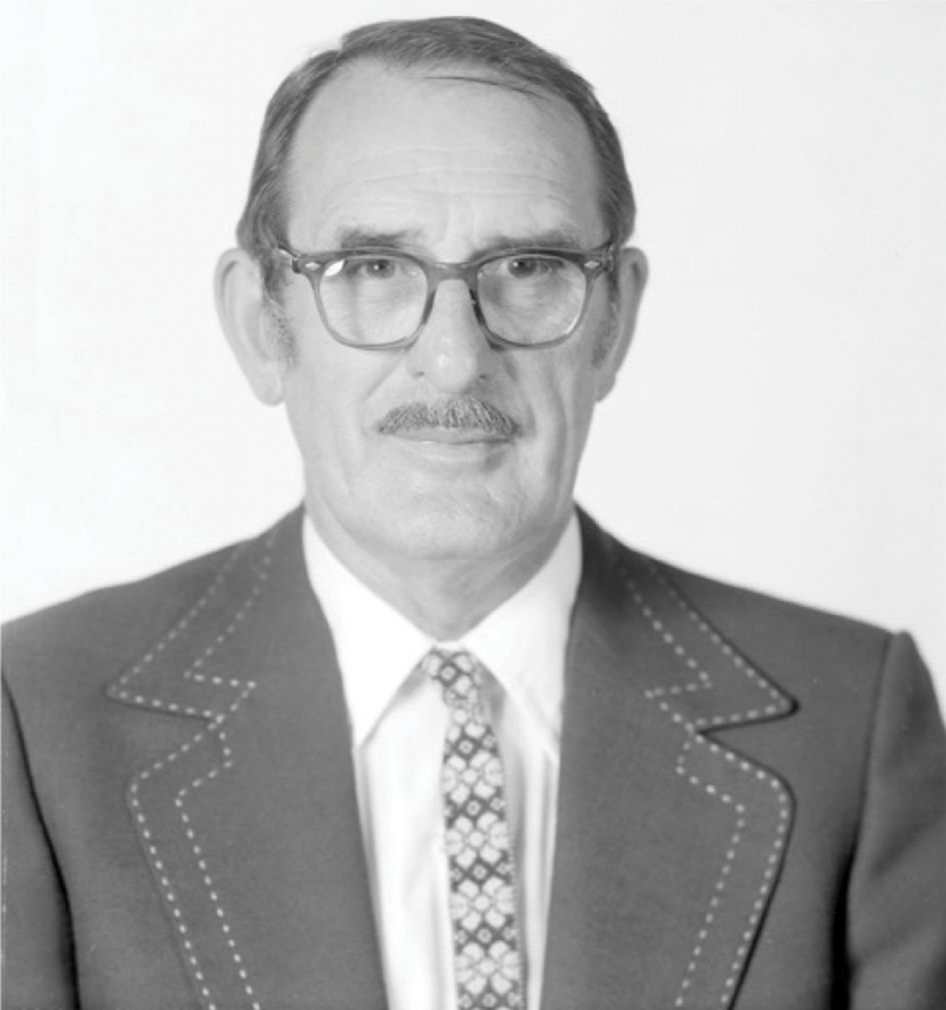
Integral figures in Q fever vaccine development: Dick Ormsbee (114). Photos courtesy of the National Institute of Allergy and Infectious Diseases.

Knowledge of Q-58A was used to develop Q-VAX through a collaboration between Richard Ormsbee at RML and Barrie P. Marmion (Fig. 4), Division of Medical Virology, Institute of Medical and Veterinary Sciences in Adelaide, South Australia (15, 110, 111). In 1979, Marmion expressed concern about the high incidence of Q fever among abattoir workers in Australia. He realized that an Ormsbee-type Q fever vaccine would be useful for this occupational group. He confirmed that a then Australian government-owned company, Commonwealth Serum Laboratories (CSL), now a private company, had the ability to make the vaccine. CSL previously made a Q fever vaccine based on the Nine Mile strain for use by its employees when preparing diagnostic reagents for serological diagnosis of Q fever (phase I and phase II antigens). Ormsbee arranged for the Henzerling strain of *C. burnetii*, then stored at Fort Detrick, to be sent to Dr. Brian Feery, a colleague of Marmion and a medical microbiologist working at CSL in Melbourne. This shipment was approved by the commanding officer at Fort Detrick and was sent to Australia via the Australian Scientific Attaché in Washington, DC, using an Australian Diplomatic Pouch toward the end of 1980. CSL began growing the vaccine in embryonated chicken eggs in early 1981. Ormsbee visited CSL later in 1981 and advised Feery on the finer points of preparing the vaccine, including maximizing purity from egg proteins. The vaccine, named Q-VAX, consisted of a formalin-inactivated Henzerling strain in phase I (110). During the June 1981–December 1988 trial period, over 4,000 individuals from four different abattoirs were vaccinated. The vast majority of vaccinees were plant workers, but frequent visitors (considered at risk) were also vaccinated. Only two serious inoculation site reactions were noted. Q-VAX was considered safe and nearly 100% efficacious. Based on this study, the vaccine was considered effective if administered 13 or more days before natural exposure to *C. burnetii*. Q-VAX was licensed for use in Australia in March 1989 by the Department of Community Services and Health in Canberra (111, 112). Infrequent vaccine failure was associated with vaccination during the incubation period when the vaccine had insufficient time to trigger significant cell-mediated immunity and thwart disease manifestation (113). Q-VAX is still widely used in Australia, which is the only country with a licensed human vaccine against Q fever. At-risk individuals have traveled to Australia simply to receive Q-VAX.

## CROSS-PROTECTION OF Q FEVER VACCINES

Several genomic typing schemes demonstrate genetic heterogeneity between *C. burnetii* strains. For example, eight genomic groups have been established by microarray-based, whole-genome comparisons, plasmid content, and restriction fragment-length polymorphisms (97, 115, 116). These groups differ in as many as 200 genes and can display notable differences in animal models of infection (95, 117). Genetic diversity also correlates with the production of structurally and antigenically unique LPS *O*-antigens (118). However, full-length *O*-antigen heterogeneity does not appear to impact vaccine efficacy as, to our knowledge, studies with vaccinated animals all show protection against heterologous challenge (117). This observation suggests conservation of critical protective epitopes of LPS and other immunogenic components between genomic groups. This may be due to polysaccharide origin in the bacterial cell wall, subjecting the molecule to less immunological selective pressure. Human vaccination also illustrates cross-protection. Vaccination with laboratory experimental vaccines was largely universal in protection against Q fever in laboratories studying distinct strains. Q-VAX is composed of the Henzerling, genomic group 2 strain and was isolated from guinea pigs in March of 1945 from the blood of an American soldier stationed in Italy (45). Henzerling was also the strain used in the US army vaccine. Although the original Australian isolate sent to the USA has now apparently been lost and was not typed, predominant strains currently circulating in Australia are all genomic group 4 and appear to have pathogenic potential. Thus, the pronounced efficacy of Q-VAX appears to be conserved regardless of the challenge strain genomic group.

## CONTEMPORARY VACCINE STRATEGIES AND CONCLUSIONS

The recent large Q fever outbreak in the Netherlands (119) and continued infections in at-risk groups, such as veterinarians (6, 120) and US military and allies returning from endemic areas, such as Iraq (7, 11), have stimulated interest in a new generation of Q fever vaccines that lack reactogenicity. The availability of genome sequences and recombinant DNA technology has allowed for the production of recombinant proteins and has led to many subunit vaccine studies. From these studies, several potential protective protein antigens have been identified (121–124). In one case, purified native proteins exhibited protective capacity (121). However, this has not been demonstrated for recombinant proteins, suggesting that antigenic epitopes on natively conformed proteins may be needed for protection or that native protein preparations used in these early studies were contaminated with small amounts of additional protective antigen, most likely LPS. Interestingly, Li et al. (124) showed that recombinant rP1-HspB fusion protein, but not P1 or HspB alone, induces protective immunity. Collectively, these data suggest multiple protein immunogens may be desirable in protein-based subunit vaccines, such as in Fratzke et al. (125). Several vaccine candidates have displayed protective efficacy and reduced reactogenicity in a guinea pig infection model compared to WCV; however, protection did not reach WCV levels when using splenomegaly as a representative endpoint.

Recent alternative vaccine approaches include soluble bacterial extract-based vaccines, which demonstrate protection in a guinea pig model of pulmonary *C. burnetii* infection (126). Furthermore, the Q-Vaxcelerate initiative has proposed an interdisciplinary approach to vaccine development, including innovative technologies such as mass cytometry and RNA replicon generation (127). Given the known protective efficacy of phase I LPS, several LPS-based vaccines have been developed, yielding promising results (128). More recently, a vaccine consisting of only purified phase I Nine Mile strain cell wall polysaccharide (free of all lipids, proteins, and nucleic acids) covalently linked to tetanus toxoid (to enhance immunogenicity) has been developed in Australia (129). Additional vaccine development strategies include modified WCV (74, 130) and vaccines targeting livestock. Current strategies are discussed in detail by Long (131) and Sam et al. (132).

Beyond *de novo* vaccine development, recent efforts have been made to better characterize mechanisms underlying WCV reactogenicity. Animal models of post-vaccination hypersensitivity (PVH) have been utilized by many groups to this end. For example, Gregory et al. (126) and Long et al. (74) have described PVH models in guinea pigs to screen vaccine candidates. Furthermore, murine PVH models have been introduced by Binette et al. (133) and Fratzke et al. (134), which allow for more detailed mechanistic interrogation of the PVH response. Indeed, Fratzke et al. recently described molecular mechanisms of PVH in a murine model, implicating cellular immunity and antigenic persistence in the severity of the PVH response (135). Further studies are needed to characterize PVH mechanisms in more physiologically relevant models of human disease. Overall, modern efforts to characterize the PVH response have illuminated important targets for reducing vaccine reactogenicity; however, continued efforts are critical to complement both recent and historical findings.

Development of a safe and effective vaccine for Q fever continues to be a worldwide research focus. The rich history of this pursuit provides a narrative of persistence, ingenuity, and scientific advancement that has informed modern experimentation. Unfortunately, this early research is often omitted from modern literature, risking the possibility that critical context, insights, and perspectives could be lost. A prime example of the value of historical information relates to the phenomenon of *C. burnetii* antigenic reversibility, which Stoker and Fiset described in 1956. Briefly, repeated passage in egg yolk causes phase I conversion to phase II and vice versa during passage in animals (80). Despite the generation of a clonal, phase II LPS-expressing strain of *C. burnetii* thought to be immune to phase variation, this phenomenon was feasible. Thus, this lost notation was demonstrated again by Long et al. (105), described as LPS elongation and attributed to a slipped strand mutation in *cbu0533* using modern molecular biological techniques. This advancement was only possible due to investigations related to the critical role of LPS in Q fever vaccine protective efficacy. Furthermore, the development of Q-VAX is an excellent example of how interdisciplinary collaboration can drive progress. These are important considerations moving forward in the continued pursuit of an effective and safe vaccine. As Stanley and Susan Plotkin stated in a seminal review of vaccine development history, “It is said that only those who have seen the beginning of things can understand the present” (136).
